# Optimization and enhancement of textile reactive Remazol black B decolorization and detoxification by environmentally isolated pH tolerant *Pseudomonas aeruginosa* KY284155

**DOI:** 10.1186/s13568-018-0616-1

**Published:** 2018-05-21

**Authors:** Rasha A. Hashem, Reham Samir, Tamer M. Essam, Amal E. Ali, Magdy A. Amin

**Affiliations:** 1 Department of Microbiology and Immunology, Faculty of Pharmacy, Cairo University, Kasr El-Aini St., Cairo, 11562 Egypt; 2 Department of Pharmaceutical Microbiology, Faculty of Pharmaceutical Sciences and Pharmaceutical Industries, Future University, Cairo, 11787 Egypt

**Keywords:** Bio-decolorization, Bioinformatics, Detoxification, Optimization, Remazol black B

## Abstract

Azo dyes are complex derivatives of diazene used in food and textile manufacture. They are highly recalcitrant compounds, and account for severe environmental and health problems. Different strains of *Pseudomonas* species were isolated from textile wastewater effluents. The bioconversion of Remazol black B (a commonly used water soluble dye) by *Pseudomonas aeruginosa* was observed in static conditions. The bio-decolorization process was optimized by a multi factorial Plackett–Burman experimental design. Decolorization of 200 mg L^−1^ reached 100% in 32 h. Interestingly, the presence of yeast extract, magnesium and iron in the culture media, highly accelerated the rate of decolorization. Moreover, one of our isolates, *P. aeruginosa* KY284155, was kept high degradation rates at high pH (pH = 9), which represents the pH of most textile wastewater effluents, and was able to tolerate high concentration of dye up to 500 mg L^−1^. In bacteria, azo-dye degradation is often initiated by reductive azo compound cleavage catalyzed by azo-reductases. Three genes encoding azo-reductases, *paazoR1, paazoR2* and *paazoR3,* could be identified in the genome of the isolated *P. aeruginosa* stain (B1). Bioinformatics analyses of the *paazoR1, paazoR2* and *paazoR3* genes reveal their prevalence and conservation in other *P. aeruginosa* strains. Chemical oxygen demand dramatically decreased and phyto-detoxification of the azo dye was accomplished by photocatalytic post treatment of the biodegradation products. We suggest applying combined biological photocatalytic post treatment for azo dyes on large scale, for effective, cheap decolorization and detoxification of azo-dyes, rendering them safe enough to be discharged in the environment.

## Introduction

Textile wastewater usually contains a large variety of dyes and chemicals additives used in the dyeing process, such as heavy metals, soda ash, caustic soda and acetic acid. Pollution with these dyes represents an important environmental challengeto the textile industry (Bansal and Kanwar 2013). Azo dyes are the most commonly used textile dyes, containing one or more azo groups attached to aromatic groups. They are difficult to biodegrade aerobically because of their chemical stability. Since oxygen is a more efficient electron acceptor than azo dyes, their aerobic bacterial treatment under shaking conditions is less efficient than aerobic treatment under static conditions or anaerobic ones (Stolz 2001; Mantzavinos and Psillakis 2004). More than 100,000 dyes are used in the textile industry and more than 700,000 tons of commercial dyes are produced annually (Lucas et al. 2007). About 10–15% of these dyes is discharged into textile wastewater effluent, which not only creates serious environmental hazards, but also renders textile wastewater aesthetically unacceptable. In addition, azo dyes themselves are toxic and highly carcinogenic. Consequently, it is important to treat textile wastewater before discharging it to the environment (Sudarjanto et al. 2006; Pratum et al. 2011). Reactive azo dyes are highly recalcitrant to conventional methods (biological methods) used in wastewater treatment because of the presence of strong electron-withdrawing groups that give them stability against bacterial degradation (Lucas et al. 2007; Gregorio et al. 2010). Most azo dye degrading microorganisms cleave the azo bond(s), which subsequently generates colourless aromatic amines. These amines are toxic products, but could be metabolized under aerobic conditions to less toxic ones (Mohanty et al. 2006).

Azo-reductases, found in bacteria, catalyse the degradation of azo dyes through the reductive cleavage of azo groups (–N=N–). In presence of an electron donor, azo dyes are transformed into colourless aromatic amines (Zimmermann et al. 1982). Azo-reductases are classified into three main groups based on their structure: Group I, the polymeric flavin-dependent NADH-preferred azo-reductases; Group II, the polymeric flavin-dependent NADPH-preferred azo-reductases and Group III, the monomeric flavin-free NAD(P)H-preferred azo-reductases (Nakanishi et al. 2001; Chen et al. 2010; Feng et al. 2012).

Since biological treatment alone does not always provide satisfactory results for industrial wastewater, the industry makes use of chemical treatment, such as an advanced oxidation process (AOP). This process generates non-specific highly reactive hydroxyl free radicals using either ultra violet radiation, TiO_2_, ozone, Fenton reaction with hydrogen peroxide or Fenton oxidation, which are highly efficient for recalcitrant wastewater treatment. It has advantages over other treatments of being a low- or non-waste-generating technology, of high oxidation power and highly destructive against organic pollutants. On the other hand, AOP is relatively expensive compared to biological methods. Thus, the combination of both biological and AOP allow eliminating of degradable pollutants of wastewater in a cost-effective and time-saving manner (Oller et al. 2011).

In this study, we isolated and identified pH-tolerant *Pseudomonas* species that are able to decolorize Remazol black B at high pH which characterizes most textile effluents. For further characterization, we applied molecular and bioinformatics analyses to assess the sequence conservation of the genes encoding azo-reductases (*paazoR1, paazoR2 and paazoR3*) possibly involved in the bioconversion process. By applying the Plackett–Burman experimental design, we implemented and assessed the optimum reaction conditions for decolorization to enhance the decolorization and detoxification of Remazol black B. Such optimization highlighted the importance of yeast extract as a co-substrate. Finally, we adopted a post AOP treatment to achieve higher decolorization and detoxification rates for the chosen dye.

## Materials and methods

### Microorganisms

Bacterial isolates were collected from various sewage and textile effluents in Cairo and Giza governments. Ten isolates were identified biochemically (using API 20 NE system, Biomerieux). The isolate with the highest decolorization percentage (B1) was selected for further molecular identification using the 16S rRNA sequence analysis technique. Two universal primers F-5′ACG CGT CGA CAG AGT TTG ATC CTG GCT-3′ and R-5′GGA CTA CCA GGG TAT CTA AT-3′ were used, and the GenBank database (NCBI, USA) was then used to search for 16S rRNA sequence similarities (El-Rakaiby et al. 2012).

### Medium and culture condition

The basal medium used was mineral salt medium (MSM) containing 4 g K_2_HPO_4_; 4 g KH_2_PO_4_; 2 g (NH_4_)_2_SO_4_; 0.5 g MgSO_4_·7H_2_O; 0.01 g CaCl_2_; 0.01 g FeSO_4_·7H_2_O per litre of distilled water. Erlenmeyer flasks of 250 mL volume, containing 100 mL MSM supplemented with 0.1% glucose and 0.4% yeast extract and 50 mg L^−1^ dye was used to carry out most of the experiments unless otherwise stated.

### Bio-decolorization study and UV–visible spectral analysis

Mineral salt medium containing 50 mg L^−1^ of Remazol black B was supplemented with 0.4% yeast extract and 0.1% glucose. After inoculation with 5 mLs obtained from the overnight culture of each isolate, the medium was incubated under static conditions (no shaking) at temperature 30 °C and pH 7. After 24 h of incubation the absorbance and decolorization percentage were determined for this purpose aliquots of 5 mLs of the cultures were centrifuged at 1400*g* for 10 min. The absorbances of supernatant filtrates were measured at ƛmax 597 nm by using UV–visible spectrophotometer (Shimazu UV). The decolorization activity was expressed as decolorization percentage, and the un-inoculated medium was used as a negative control. The decolorization percentage was calculated as follows:

$$ {\text{Decolorization }}\%  = \frac{{initial\;absorbance - observed\;absorbance}}{{initial\;absorbance}} \times 100. $$

The decolorization of Remazol black B was studied at different time intervals. For that purpose, the isolate was grown in the same medium and under the same conditions used in the bio-decolorization study.

### Effect of pH

The effect of different pH values (5, 6, 7, 8 and 9) of the culture media on the decolorization was tested at constant concentration of Remazol black B (50 mg L^−1^) in 50 mL MSM supplemented with 0.1% glucose and 0.4% yeast extract under static conditions and of temperature 30 °C for 24 h.

### Effect of static and shaking conditions

The effect of static and shaking conditions on decolorization was tested using a dye concentration of 50 mg L^−1^ in 50 mL MSM supplemented with 0.1% glucose and 0.4% yeast extract at pH 7 and 30 °C under static and shaking conditions. Shaking was carried out at 180 rpm.

### Crude enzyme extraction

The bacterial cells were grown in MSM media supplemented with 0.1% glucose, 0.4% yeast extract and 50 mg L^−1^ dye to enhance the production of azo-reductase enzyme. The bacterial cells grown in absence of dye were considered as control. Both cells were harvested by centrifugation at 12,000 rpm for 10 min, then the cells were washed three times by phosphate buffer (100 mM, pH 7.4) and suspended in 5 mL of the buffer. The cells were lysed by sonication at 200 amp, giving 99 stroke, each of 3 s with 7 s interval at 4 °C, then centrifuged at 5000 rpm for 30 min at 4 °C. The supernatants were then used for further analyses. The total protein concentration was determined by Bradford method (Zhao et al. 2014).

### Azo-reductase activity

Azo-reductase activity was determined by monitoring the dye reduction in 270 s. The assay mixture consisted of; 600 µL phosphate buffer (50 mM, pH 7.5), 100 µL dye (50 mM), 100 µL the crude enzyme and 200 µL NADH. One unit of azo-reductase activity was defined as 1 μg of dye reduced per milligram protein per minute (Du et al. 2011).

### Screening of azo-reductase genes

Primers for azo-reductase genes (*paazoR1, paazoR2 and paazoR3*) were designed (http://eu.idtdna.com/PrimerQuest/Home) according to the gene sequences in *P. aeruginosa* PAO1 (reference strain which possess azo-reductase genes). The primers were used to amplify these genes the genomic DNA of *P. aeruginosa* (B1 isolate with highest decolorization potential). Primers were checked for their specificity by blasting their sequences against NCBI non-redundant sequence database. The sequences of the chosen primers sets for azo-reductase genes are as follows: for *paazoR1* 89F 5ʹTTTCCTGGCGGCCTATC3ʹ, 485R 5ʹGGTTCATCGCCTGGTTCT3ʹ, *paazoR1* 31F 5ʹTTGCAGTGCATGCCAGTC3ʹ and 535R 5ʹCCTCGTCGATGCCGATGAAA3ʹ, for *paazoR2* 34F 5ʹGCATATCGATTCCAGCATCCTC3ʹ and 251R 5ʹGGAACTCTTCCAGCACTTCTTC3ʹ and for *paazoR3* 162F 5ʹGCCGCACCTCGATGAATTA3ʹ and 616R 5ʹAAAGGGAGGTGTCGGTTTC3ʹ.

### Bioinformatics analyses

The predicted protein sequences of the azo-reductase of B1 were blasted against the NCBI protein database sequences using BlastP (Altschul et al. 1990). Different azo-reductase protein sequences from various microorganisms were aligned against those of B1 *P. aeruginosa* using the alignment tool of CLC Main Workbench 5 (CLC Bio, Aarhus, Denmark). Finally, this alignment was used to construct a phylogenetic tree using the same previously mentioned software.

### Multi-factorial design

Biodegradation optimization of Remazol black B by the selected isolate was attempted using Placket–Burman design, where eleven independent factors were included. Eight factors affecting the medium composition were assessed, namely; Glucose concentration, Sodium acetate, Yeast extract, Sodium Nitrate, iron concentration, Magnesium concentration, EDTA and NaCl. Two factors affecting the environmental condition namely; pH and temperature, in addition to dye concentration was also included in this design (Table 1).

**Table 1:** Experimental range and levels of independent factors in the Plackett–Burman design

Factor	Unit	Symbol	Levels		
			Low − 1	Center 0	High + 1
Glucose	g L^−1^	C	0	1	5
Yeast extract	g L^−1^	N	0	4	8
NaCl	g L^−1^	Salt	0.1	0	2
Sodium nitrate	g L^−1^	NO_3_	0.1	0	2
Ferrous sulfate FeSO_4_·7H_2_O	g L^−1^	Fe	0	0.01	0.5
Magnesium sulfate MgSO_4_·7H_2_O	g L^−1^	Mg	0	0.5	1
Initial pH	pH	pH	5	7	8
Temperature	°C	Temp	25	30	37
Dye concentration	mg L^−1^	Conc	25	50	250
EDTA	mM	EDTA	0.1	0	2
Sodium acetate	g L^−1^	Acetate	0.1	0	1

For each factor, a high (+) and low (−) levels were tested (Hassan and Essam 2015). The placket–Burman design matrix included 12 runs of various combinations of levels of the assessed factors in addition to 13th run under control conditions using the following medium ingredients and incubation conditions (4 g K_2_HPO_4_; 4 g KH_2_PO_4_; 2 g (NH_4_)_2_SO_4_; 0.5 g MgSO_4_·7H_2_O; 0.01 g CaCl_2_; 0.01 g FeSO_4_·7H_2_O per litre of distilled water 1 g glucose and 4 g yeast extract at pH 7 and 30 °C) (Table 2).

**Table 2:** The Placket–Burman design matrix representing the coded values for independent factors and values of measured response

Run	Factors											Response
	Glu	Yeast	NaCl	NO_3_	Fe	Mg	pH	Temp	conc	EDTA	Acetate	% decolorization
1	+	–	+	–	–	–	+	+	+	–	+	8.65
2	+	+	–	+	–	–	–	+	+	+	–	2.71
3	–	+	+	–	+	–	–	–	+	+	+	11.78
4	+	–	+	+	–	+	–	–	–	+	+	5.87
5	+	+	–	+	+	–	+	–	–	–	+	46.91
6	+	+	+	–	+	+	–	+	–	–	–	88.22
7	–	+	+	+	–	+	+	–	+	–	–	29.42
8	–	–	+	+	+	–	+	+	–	+	–	32.32
9	–	–	–	+	+	+	–	+	+	–	+	21.04
10	+	–	–	–	+	+	+	–	+	+	–	20.04
11	–	+	–	–	–	+	+	+	–	+	+	66.45
12	–	–	–	–	–	–	–	–	–	–	–	0.88
13	0	0	0	0	0	0	0	0	0	0	0	65.2

(0) represents the original concentration of each components under the control conditions, (−) represents the low level and (+) represents the high level for each factors

*Glu* glucose concentration, *Yeast* yeast extract concentration, *NO*_*3*_ sodium nitrate, *Fe* iron concentration, *Mg* magnesium concentration, *conc* dye concentration

All runs were done in triplicates, where a total of 39 runs were applied. The response measured after 24 h of incubation was the decolorization of the dye calculated from the difference between initial and observed absorbance at the λ_max_ (597 nm).

### Combined biological-photochemical process

The combined biological-photochemical treatment of Remazol black B was tested by inoculating the medium supplemented by the dye (50 mg L^−1^) with B1 isolate and incubating it for 24 h. This was followed by photocatalytic treatment for another 24 h using UVA lamp (40 W, 56 cm) and/or UVC lamp (15 W, 40 cm) in presence of 1 g L^−1^ of TiO_2_ as a photo-catalyst. The mixture of TiO_2_ and dye solution was sonicated for 1 min to obtain a homogenous suspension. Then 5 mL of the suspension was transferred into 9 × 1 cm screw caped glass tubes at shaking conditions of 200 rpm at room temperature. The tubes on the shaker were exposed to UV irradiation at distance of 10 cm from the UV lamp. Samples withdrawn from these tubes were centrifuged at 1400*g* for 10 min and the supernatant was filtered by syringe filter 0.45 mm then analysed to calculate the remaining dye concentration.

### COD and phyto-toxicity assessments

COD measurements were performed for both biological and photocatalytic treatments. COD measurements were carried out using a closed reflux titrimetric method (American Society for Testing and Materials 1995).

Phytotoxicity was assayed by adding five seeds of *Lepidium sativum* onto a 5.5 cm filter paper placed in a Petri dish filled with 2 mL of the sample, where the samples represented the media supplemented with the dye after different treatments. The samples were diluted two times, centrifuged for 10 min at 1400*g* (Hettich EBA20, UK) and the supernatants were applied to the plates. The plates were then covered and incubated in complete darkness for 3 days. Each test was performed in triplicate, and phytotoxicity percent was calculated as the ratio of the average reduction in stem length of 15 test seeds to the average of reduction in stem length 15 control seeds (treated with tap water or MSM) this test was carried out against a positive control were the seeds were subjected to untreated media supplemented by the dye (Essam et al. 2006).

### Statistical analyses

Statistical analyses and graphical presentation of data for multifactorial design was performed using Minitab software (Version 17). Graph Pad Prism 5 software was used for most of the statistical analyses, e.g., One way ANOVA and Dunnett’s Multiple comparisons post-test at P-value < 0.05.

## Results

### Identification of isolated biodegrading strains

**Fig. 1:**
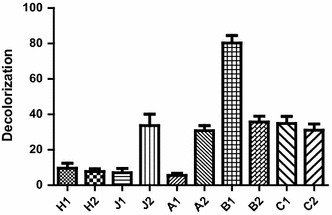
Decolorization of Remazol black B by different isolates at concentration 50 mg L^−1^ after 24 h incubation in MSM media supplemented with 0.1% glucose and 0.4% yeast extract, at pH 7 and 30 °C under static conditions

Ten bacterial isolates capable of decolorizing Remazol black B were isolated from various sewage and textile effluents in Cairo and Giza governments. They were identified biochemically using API 20 NE system as follows: four isolates were identified as *Pseudomonas aeruginosa*, three isolates as *Pseudomonas putida*, one as *Pseudomonas fluoresences,* one as *Pseudomonas* spp. and one as *Alcaligenes xylosoxidans*. When all ten isolates were studied for Remazol black B decolorization, isolate B1 (*P. aeruginosa*) was found to have the highest decolorization (Fig. 1) and was thus selected for further molecular identification.

### Molecular identification of isolated biodegrading strain

Isolate B1 used in this study was identified on the basis of 16S rRNA gene sequencing. The closest hit in GenBank database was found to be *Pseudomonas aeruginosa* with 99% sequence identity. The sequence was submitted to Genbank with an accession number of KY284155. This isolate was also deposited in the publicly accessible culture collection belonging to WDCM, Egypt Microbiological Culture Collection, and has assigned a number (EMCC1801).

### Azo-reductase activity

**Fig. 2:**
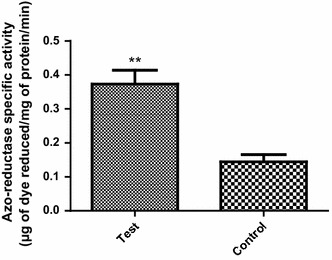
Specific azo-reductase activity of isolate B1 when the cells were grown in the presence of the dye (test) as opposed to those grown in absence of the dye (control). The data represent means of triplicate experiments ± S.E. Decolorization by test cells is significantly different than by control cells at *P* < 0.01 (two-tailed t-test)

The enzymatic activity of azo-reductase significantly increased when the cells were grown in presence of the dye in comparison to those grown in the absence of the dye (Fig. 2).

### Screening of azo-reductase genes

Three azo-reductase genes were amplified from genomic DNA of *P. aeru*ginosa (isolate B1) by PCR using specific sets of primers. The screened genes were *paazoR1, paazoR2 and paazoR3.* Primers produced positive PCR results for both isolate B1 and *P. aeruginosa* PAO1 reference strain. Product sizes were 522, 239 and 473 bp for *paazoR1, paazoR2 and paazoR3* genes, respectively.

### Bioinformatics analyses

Azo-reductase gene sequences of isolate B1 possess high similarity to that of PAO1 reference strain (sequence similarity ranged from 97 to 99%).

**Fig. 3:**
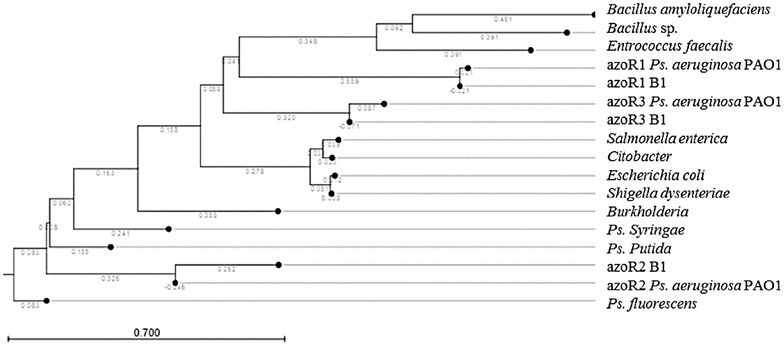
phylogenetic tree of *Pseudomonas aeruginosa* (B1 isolate), *Pseudomonas aeruginosa* PAO1 (reference strain) and other bacterial species.*Pseudo* Phylogentic tree was generated using azo-reductase protein sequences showing the evolution distances, represented by numbers written on branches, from hypothetical ancestors represented by nodes between the isolated *Pseudomonas aeruginosa* and different bacteria showing high relatedness between different strains of *Pseudomonas aeruginosa* and high variations when compared to other bacterial species. The tree was generated by CLC workbench 5 software

A phylogenetic tree was constructed from the azo-reductase protein sequences (Fig. 3). The tree indicated the evolutionary distances from hypothetical ancestors represented by nodes between proteins from isolate B1 (*P. aeruginosa*) and azo-reductases from different bacteria. High relatedness among the proteins of different *P. aeruginosa* strains can be observed, while the sequence variation increased when compared to other bacterial species.

### Time course of decolorization of Remazol black B by *Pseudomonas aeruginosa* (B1 isolate)

**Fig. 4:**
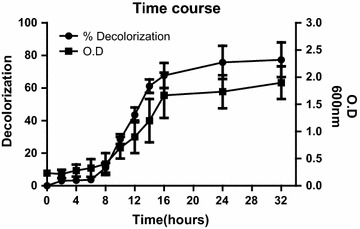
Time course of decolorization of Remazol black B by isolate B1 *(Pseudomonas aeruginosa*). Decolorization and O.D_600_ of samples taken after 0, 2, 4, 6, 8, 16, 24 and 32 h were plotted against time. The data presented are the mean of 3 independent experiments and the error bars represent the standard deviation (SD)

Decolorization of Remazol black B by isolated B1 strain started after 8 h since cultivation, with 11% decolorization, and reached 67.7% within 24 h (Fig. 4).

### Optimization of Remazol black B decolorization using multifactorial design (Plackett–Burman design)

B1 strain decolorization was optimized following a multifactorial design (Plackett–Burman design) and the E-values of the measured response were recorded as decolorization of Remazol black B after 24 h (Table 2).

Experiments were performed as combinations of factors shown in Table 2. The corresponding response of dye decolorization varied from 0.88 to 88.22%.

**Fig. 5:**
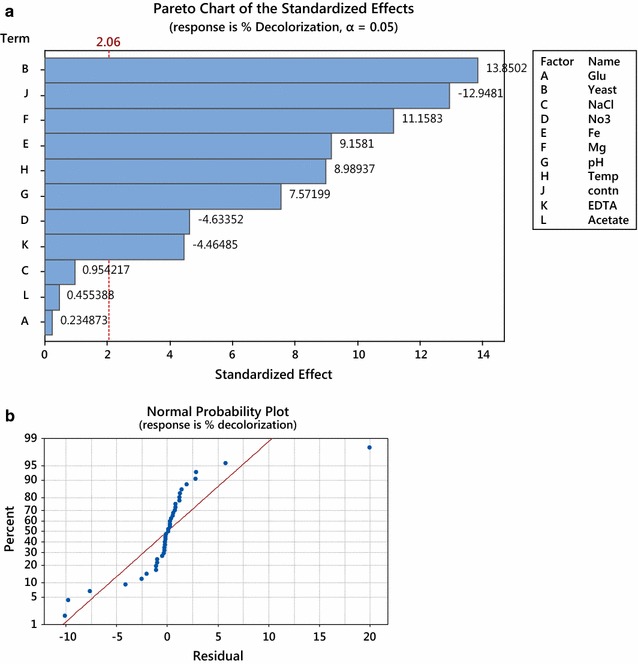
**a** Pareto charts ranking the standardized effects of the tested factors. The vertical line in chart represent a reference line, any factor that extends past this line is of significant effect at α = 0.05 (significance level). E-value (the standardized effect of each factor) was calculated using Minitab software (version 17). The magnitude of the E-value of the tested factor indicated its significance in affecting the response, while the positive or negative sign of the E-value was an indication of its positive or negative influence on the responses, respectively. **b** Normal probability plot of residuals for isolate B1. The straight line in the graph represents the mathematical regression equation (which determines the expected data), while the dots in the plots represent the actual observed data. As shown in figure the dots generally formed a line consistent with regression line, therefore the residuals (the difference between observed data and expected data) are considered normally distributed

The standardized effect of each factor (E-value) was calculated using Minitab software (version 17). The E-value magnitude of the tested factor indicated its effect or its significance in affecting the response, while the positive or negative sign of the E-value is an indicative of its positive or negative influence on the responses, respectively. The standardized effects magnitude and significance of each factor are shown in Pareto charts (Fig. 5a). Most factors significantly affected the decolorization. These included yeast extract, dye concentration, magnesium, iron concentration, temperature, pH-value, nitrates and the presence of EDTA. On the other hand, glucose, sodium acetate and sodium chloride were not significant factors. The order of significance of these variables affecting the dye decolorization is shown (Fig. 5a).

Normal probability plot of residuals was used to examine the goodness of the model fit (Fig. 5b). The straight line in the graph represent the mathematical regression equation (which determines the expected data), while the dots in the plots represented the actual observed data. The dots generally formed a line consistent with regression line, therefore the residuals were considered normally distributed.

Run no. 13 represented the control medium composition under the control conditions. Using this run, isolate B1 showed a decolorization of 65.2%. Isolate B1 showed remarkable and significant decrease in decolorization in all runs while runs no. 6 showed significant increase in decolorization and run no 11 showed no significant increase in decolorization.

**Fig. 6:**
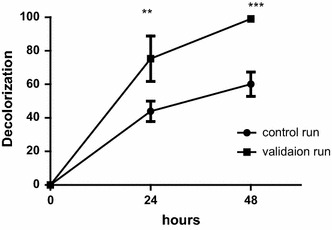
Decolorization of Remazol black B by isolate B1 in the control run with media composition and under conditions before optimization [4 g K_2_HPO_4_; 4 g KH_2_PO_4_; 2 g (NH_4_)_2_SO_4_; 0.5 g MgSO_4_·7H_2_O; 0.01 g CaCl_2_; 0.01 g FeSO_4_· 7H_2_O per litre of distilled water 1 g glucose and 4 g yeast extract at pH 7 and 30 °C] and the validation runs using the predicted optimal medium [4 g K_2_HPO_4_; 4 g KH_2_PO_4_; 2 g (NH_4_)_2_SO_4_; 1 g MgSO_4_·7H_2_O; 0.01 g CaCl_2_; 0.5 g FeSO_4_·7H_2_O, 1 g glucose and 8 g yeast extract per litre of distilled water at pH 8 and temperature 37 °C], *means that there is significant difference from the control run at P-value < 0.05 (One way ANOVA, paired t test), increase in number of (*) is proportional to the significance difference

The higher decolorization in run no. 6 was achieved by the following medium composition; 4 g K_2_HPO_4_; 4 g KH_2_PO_4_; 2 g (NH_4_)_2_SO_4_; 1 g MgSO_4_·7H_2_O; 0.01 g CaCl_2_; 0.5 g FeSO_4_·7H_2_O, 5 g glucose and 8 g yeast extract per litre of distilled water at pH 5 and temperature 37 °C. Additional validation runs were adopted using the predicted optimal medium (medium composition of run no. 6) with 1 g L^−1^ as glucose was not a significant factor on the decolorization process and under the predicted optimal pH value (8) as pH significantly increased the decolorization process. Validation runs showed a significant increase in decolorization compared to other runs (Fig. 6).

**Fig. 7:**
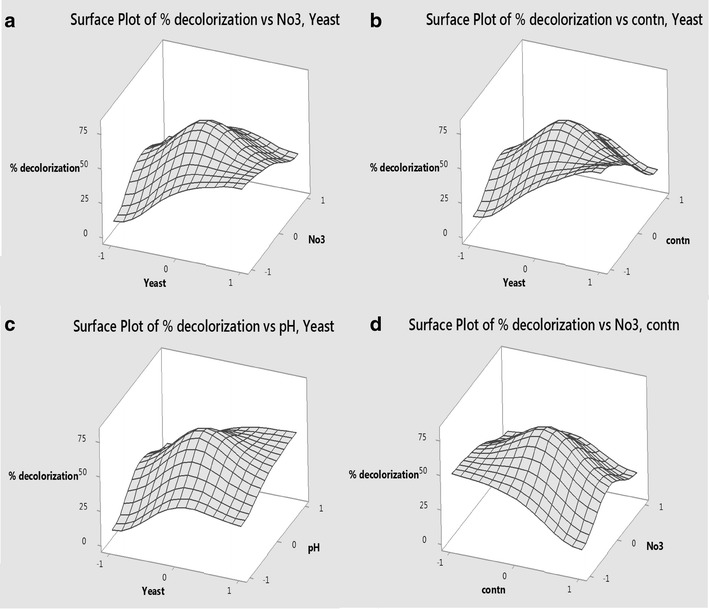
Three dimensions (3D) surface plots showing the relationship between the most significant factors and their effect on the decolorization of the dye. **a** 3D surface plot for the effect of yeast extract and nitrate concentration on the decolorization of Remazol black B by isolate B1. **b** 3D surface plot for the effect of yeast extract and dye concentration on the decolorization of Remazol black B by isolate B1. **c** 3D surface plot for the effect of yeast extract concentration and pH on the decolorization of Remazol black B by isolate B1. **d** 3D surface plot for the effect of dye and nitrate concentration on the decolorization of Remazol black B by isolate B1

Higher concentration of yeast extract (8 mg L^−1^) and lower dye concentration (25 ppm) had the highest significant effect on the percent of decolorization. Increasing magnesium and iron concentration to 1 and 0.5 mg L^−1^, respectively, also significantly affected the decolorization. To investigate the interactive effect of the two factors on the dye decolorization, surface plots were drawn from data in Table 2. The interaction between yeast extract and nitrate concentration showed that upon decreasing nitrate concentration and increasing yeast extract the decolorization of Remazol black B by B1 strain was increased. The highest decolorization was achieved at high yeast extract concentration (8 g L^−1^) and low nitrate concentration (0.1 g L^−1^) (Fig. 7a).

### Effect of pH on decolorization

Isolate B1 showed highest decolorization at pH 9 and there was a significant decrease in the decolorization at pH 5, but no significant change was observed at pH 6 and 8.

### Effect of static and shaking conditions on decolorization

Decolorization of Remazol black B decreased from 75.5 to 4.26% after 48 h by changing the static incubation condition into shaking condition.

### Combined biological–photochemical process

Increasing dye concentration led to decrease in the decolorization under photocatalytic treatment for 24 h (Table 3).

**Table 3:** Decolorization of Remazol black B under photocatalytic treatment for 24 h (UV/TiO_2_) at different dye concentrations

Dye concentration (mg L^−1^)	Decolorization under photocatalytic treatment (UV/TiO_2_)		
	UVA (%)	UVC (%)	UVA and UVC (%)
10	59.6	70	100
50	10.3	20.6	26.6

The sequential combination of biological-photocatalytic treatment showed a significant increase in decolorization, as it increased from 66.23 (biological only) to 100% (combined treatment) decolorization of 50 mg L^−1^ of the dye. Moreover, the inhibition in the growth of the stem of *Lepidium sativum* seeds decreased after combined biological-photocatalytic treatment reaching 0%. The COD levels were also reduced from 2069 to 389.2 mg L^−1^ with a percent reduction 81.1.

Upon using the optimized medium and conditions followed by photocatalytic treatment an increase in decolorization of higher concentrations of the dye was obtained and a reduction in the COD was achieved (Table 4).

**Table 4:** Decolorization of Remazol black B, COD reduction percent and phytotoxicity assessment using after biological and photocatalytic treatment at different dye concentrations

Dye concentration		Decolorization		COD reduction (%)	Phytotoxicity (the inhibition in the growth of the stem of *lepidium sativum*), %
		Biological treatment (%)	Photocatalytic treatment (%)		
200 mg L^−1^		92.8	100	83.2	0
500 mg L^−1^					
24 h		75.3	100	66.4	7.6
48 h		100			

## Discussion

The development of environmentally favourable, cost-effective techniques to treat azo dyes in textile wastewater have attracted a great attention. Reactive azo dyes are highly recalcitrant to conventional wastewater treatment because of the presence of strong electron-withdrawing groups giving them stability against bacterial degradation (Gregorio et al. 2010). In the present study, the characterization, optimization and the application of a biological or a photocatalytic or a combination thereof were investigated to enhance the decolorization and the detoxification of Remazol black B.

*Pseudomonas* genus possesses a particularly important role in biodegradation of azo dyes (Joe et al. 2011). In this work, different isolates of *Pseudomonas* species were isolated from textile wastewater effluents. One of the ten isolates was able to efficiently decolorize the azo dye within 24 h (Fig. 1). A number of enzymes have been demonstrated to participate in dye degradation. For example, laccases are able to decolorize azo dyes through nonspecific oxidation of aromatic rings in presence of oxygen as an electron acceptor (Chivukula and Renganathan 1995). Furthermore, fungal degradation involves oxidation of aromatic rings through lignin peroxidase (Glenn and Gold 1983). In bacteria, degradation of azo dye is often initiated by reductive cleavage of azo bond with azo-reductases (Zimmermann et al. 1982). Azo-reductases were considered as the key players in dye decolorization. This is suggested after the sharp reduction in the decolorization process under shaking conditions indicating that decoloriztion is carried out through a reduction process. Oxygen competes with azo dye for azo-reductase enzymes (Chang and Lin 2001). This result was also confirmed by the decrease in decolorization in presence of ammonium nitrate. This can be explained by the competition between nitrates and the azo dye on the bacterial enzymes (Chinwetkitvanich et al. 2000; Lourenco et al. 2001). The increased specific activity of the azo-reductase enzyme in presence of the dye suggests that the dye stimulates the induction of these enzymes and elucidates its activity in azo dye reduction (Lade et al. 2015). Moreover, the reduction pathway was insured by the molecular identification of three azo-reductase genes namely: *paazoR1, paazoR2* and *paazoR3* belonging to FMN dependent NADH azo reductase family in our strain. These genes were proven, by bioinformatic analyses, to be highly conserved in different *Pseudomonas* species, yet different enough from other bacterial strains. Such molecular conservation make them a potential stable trait and emphasizes the potential use of these isolates in bioremediation process. In summary, understanding and characterizing azo-reductase genes will lead to the systematic use of their expressed enzymes in new strategies for future textile wastewater bioremediation treatments.

Optimization of the bio-decolorization process of the dye, using multi-factorial design, is a must to reach the ultimate possible decolorization rates. From this design, several factors were proven to have high impact on the bio-decolorization process. Variation in incubation temperature dramatically affected the enzyme’s activity, which might be attributed to the loss of cell viability at higher temperature (Joe et al. 2011; Saratale et al. 2011). *Staphylococcus hominis* was able to decolorize Acid Orange in the temperature range of 30–35 °C, whereas, at 40 °C decolorization was completely inhibited (Singah 2014).

The initial dye concentration significantly affected the efficiency of the decolorization process. Some studies proved the possible toxicity of azo dyes to microorganisms involved in biodegradation. Toxicity is related to dye concentration, dye type and the blockage of active sites of azo reductase enzymes by dye molecule (Singah 2014).

The presence of yeast extract significantly affected the decolorization. This may be due to the stimulatory effect of nitrogen on cell growth which led to better azo dye degradation (Lourenco et al. 2000). Our results were consistent with that of Wang et al. (2008) who reported that nitrogen source is essential for the decolorization process. In addition yeast extract is a complex organic substrate that provides carbon, nitrogen, and growth factors, which can be used by the microbial cells as a source of electron donor for the reductive cleavage of the azo dyes. The metabolism of organic nitrogen sources regenerates NADH, which is an essential cofactor for the reduction of azo dyes by azo-reductases. Yeast extract was found to be the best nitrogen source for the decolorization of azo dyes (Khalid et al. 2008; Saratale et al. 2009; Singah 2014).

pH is also an important factor to the biological decolorization of azo dyes. In this study, the highest decolorization level was obtained at pH 9. It is possible that pH change affects the transport of dye molecules across the cell membrane, which is considered as the rate-limiting step for the decolorization (Saratale et al. 2011). The medium pH has a major effect on the efficiency of dye decolorization because of the dependence of the enzymatic activity on the pH. Although most Azo-reductase enzyme optimum pH is 7, some alkali-thermostable azo-reductase from *bacillus* sp strain SF showed highest decolorization and optimum pH within range from pHs 8 to 9 (Kandelbauer et al. 2004). Reactive azo dyes lose hydrogen ions under alkaline condition, which leads to the ionization of the dye, affecting its stability and facilitating its removal from solutions (Mahmoud et al. 2007). Decolorization under alkaline conditions has been generally preferred in industrial applications, as processes using reactive azo dyes are performed under alkaline conditions. The pH tolerance of decolorizing bacteria is quite important, as it makes them suitable for the commercial treatment of effluents contaminated by dye residuals (Aksu 2003), which is an exciting privilege in our B1 isolate.

Advanced oxidation processes (AOPs) generate highly non-specific reactive hydroxyl radicals, capable of destroying different organic pollutants in wastewater, but unfortunately they are expensive. In our study, increasing dye concentration decreased the dye decolorization by the photocatalytic treatment, as significant amount of UV is absorbed by dye molecules rather than the TiO_2_ particles, thus reducing the efficacy of the catalytic reaction. Furthermore, the intermediates formed upon the degradation of the dye molecules may also compete with the dye molecules for the limited adsorption and catalytic sites on the TiO_2_ particles and thus inhibit the decolorization. Such interference would be more pronounced in the presence of an elevated level of degradation intermediates formed upon increasing initial dye concentration and may need more time of irradiation which would increase the cost of the treatment. Moreover as the concentration and depth of the solution’s color increase, it will reduce the light path length and hinder its penetration (So and Cheng 2002; Karimi et al. 2011).

The combination of both biological and photocatalytic treatment enhanced the decolorization and detoxification processes. Upon applying the optimized biological conditions, the combined method was able to detoxify higher concentrations of the dye, up to 500 mg L^−1^, in 48 h. Our results agree with Shah et al. (2013) who found that biological treatment was effective in color removal, aiding the photocatalytic process to overcome the light penetration problem. Application of biological-photocatalytic process was more efficient than either single treatment alone. In addition, the combination is cost effective compared to the amount of electrical power utilized in the photocatalytic treatment alone.

Different microorganisms have been reported to decolorize azo dyes. *Providencia rettgeri* strain HSL1 was found to decolorize 100% of Reactive Blue 172 at concentration 50 mg L^−1^ with 85% COD reduction (Lade et al. 2015). *Candida tropicalis* TL-F1 was found to decolorize Acid Brilliant Scarlet GR, Acid Orange II, Acid Orange G, Reactive Brilliant Red K-2G, Reactive Yellow 3RS Red X-3B and Reactive Green KE-4BD by 97.2, 92.7, 91.1, 95.4, 94.2 and 82.6% of decolorization respectively at concentration 20 mg L^−1^ (Tan et al. 2013). *Pichia occidentalis* was found to decolorize 98% of Acid Red B at concentration 50 mg L^−1^ within 16 h (Song et al. 2017).

Photocatalytic degradation of Direct Green 6 and Reactive Orange 72 at concentration 60 mg L^−1^ using 400w UV lamp for 3 h yielded 50% decolorization (Karimi et al. 2011). Decolorization of Direct Blue 71 at concentration 100 mg L^−1^ reached 94 and 50.7% COD removal using Fenton’s oxidation process as an advanced oxidation process (Ertugay and Acar 2013).

Photocatalytic and microbial degradation using *Rhodospirillum* and under irradiation with 300 W fluorescent lamp for 5 h yielded 94% decolorization of reactive brilliant red X-3b and 84.7% COD reduction at concentration 50 mg L^−1^ (Zhang et al. 2017).

Finally, we conclude that biological and post photocatalytic treatments for textile wastewater containing azo dyes represent a promising alternative to conventional single process treatment for textile wastewater effluents with the advantages of being cheap and non-toxic. The obtained results support the efficiency of multi-factorial designs in elucidating the significance of different factors and predicting optimum conditions for the decolorization of azo dye Remazol black B by our stain that harbors an active set of azo-reductase genes.

## Abbreviations

AOPs: advanced oxidation process
COD: chemical oxygen demand
MSM: mineral salt media
